# Age as a moderating factor of treatment resistance in depression

**DOI:** 10.1192/j.eurpsy.2023.17

**Published:** 2023-04-20

**Authors:** Alexander Kautzky, Lucie Bartova, Gernot Fugger, Markus Dold, Daniel Souery, Stuart Montgomery, Joseph Zohar, Julien Mendlewicz, Chiara Fabbri, Alessandro Serretti, Dan Rujescu, Siegfried Kasper

**Affiliations:** 1 Department of Psychiatry and Psychotherapy, Medical University of Vienna, Vienna, Austria; 2 Centre Europèen de Psychologie Medicale, Université Libre de Bruxelles and Psy Pluriel, Brussels, Belgium; 3 Imperial College London, University of London, London, UK; 4 Psychiatric Division, Chaim Sheba Medical Center, Tel HaShomer, Israel; 5 School of Medicine, Free University of Brussels, Brussels, Belgium; 6 Institute of Psychiatry, Psychology & Neuroscience, King’s College London, London, UK; 7 Department of Biomedical and Neuromotor Sciences, University of Bologna, Bologna, Italy; 8 Center for Brain Research, Medical University of Vienna, Vienna, Austria

**Keywords:** treatment-resistant depression, antidepressant, sold age

## Abstract

**Background:**

Treatment-resistant depression (TRD) is an important clinical challenge and may present differently between age groups.

**Methods:**

A total of 893 depressed patients recruited within the framework of the European research consortium “Group for the Studies of Resistant Depression” were assessed by generalized linear models regarding age effects (both as numerical and factorial predictors) on treatment outcome, number of lifetime depressive episodes, hospitalization time, and duration of the current episode. Effects of age as numerical predictor on the severity of common depressive symptoms, measured with Montgomery–Åsberg Depression Rating Scale (MADRS) for two-time points, were assessed by linear mixed models, respectively, for patients showing TRD and treatment response. A corrected *p* threshold of 0.001 was applied.

**Results:**

Overall symptom load reflected by MADRS (*p* < 0.0001) and lifetime hospitalization time (*p* < 0.0001) increased with age in TRD patients but not treatment responders. In TRD, higher age was predicting symptom severity of inner tension, reduced appetite, concentrations difficulties, and lassitude (all *p* ≤ 0.001). Regarding clinical significance, older TRD patients were more likely to report severe symptoms (item score > 4) for these items both before and after treatment (all *p* ≤ 0.001).

**Conclusions:**

In this naturalistic sample of severely ill depressed patients, antidepressant treatment protocols were equally effective in addressing TRD in old age. However, specific symptoms such as sadness, appetite, and concentration showed an age-dependent presentation, impacting residual symptoms in severely affected TRD patients and calling for a precision approach by a better integration of age profiles in treatment recommendations.

## Introduction

Throughout most stages of human life, major depressive disorder (MDD) is a common and severe chronic disorder that leads to high morbidity and mortality [1]. While prevalence rates of MDD are known to vary across countries and to be sex and gender-specific [2], peak rates were often reported from late adolescence to the forties [3], while a decline was proposed in elderly patients [4]. The clinical presentation of MDD thereby shows considerable variation in symptoms and severity [5], which were partly demonstrated to be dependent on age [6]. While some studies reported less severe episodes in elderly depressed patients compared to younger adults, others raised concern for increased suicidality and unfavorable trajectories following treatment in older patients [6, 7].

Treatment-resistant depression (TRD) after adequate antidepressant trials (ADs) is challenging from a clinical perspective, being associated with a relevant disability burden and life-threatening symptoms, such as suicidality [8]. Therefore, TRD has gained significant scientific interest and a broad range of risk factors was identified, including psychiatric comorbidities such as anxiety disorders, symptom severity, and personal disease history, such as presence of suicidal and psychotic features, age of onset, recurrence, and number of episodes [9–11].

Nevertheless, age-specific factors modulating treatment outcome and specifically TRD need further investigation, as the efficacy of well-established antidepressants was shown to vary in different age groups and may be compromised in elderly depressed patients [12, 13]. Thereby, TRD may be more common with increasing age and affect more than a third of elderly patients [14]. While some antidepressant agents were specifically studied to target TRD in older MDD patients [13, 15], the role of age in TRD remained mostly inconclusive. Exploiting a large multinational European database on TRD that was built for well over two decades by the “Group for the Studies of Resistant Depression” (GSRD) [10, 16], age was previously suggested as an important moderator of treatment outcome, despite without direct association with TRD or response phenotypes [17, 18].

### Aims of the study

Following up on these results, we conducted an analysis on the impact of age as a continuous variable as well as categorical age groups on clinical profiles of treatment outcomes, response, and TRD.

## Methods

### Sample description

This analysis was performed in a multinational European sample of 1410 patients diagnosed with MDD according to DSM-IV criteria, labeled as TRD-III and collected within the scope of the research consortium GSRD to define predictors of resistance to antidepressant treatment. In- and outpatients in university and community hospitals were enrolled across 10 European counties and Israel. Diagnoses were determined with a modified version of the MINI-International Neuropsychiatric Interview 5.0.0 (MINI) and depressive symptom severity was assessed with the “Montgomery–Åsberg Depression Rating Scale” (MADRS). Further, only adult patients with a primary diagnosis of MDD that were free of severe personality disorders or any current substance abuse or addiction disorder except for nicotine and caffeine were recruited. Details of the study sample including baseline characteristics and psychopharmacological agents used have been published previously [10].

### Treatment outcomes

Treatment outcome phenotypes were classified according to the GSRD staging system, as previously described by our group, and supported by the European Medicines Agency (EMA; http://www.ema.europa.eu) [19, 20]. In short, according to the GSRD staging system TRD is defined by failing to achieve relevant improvement of at least 50% of points on a recognized depression symptom severity scale after at least two ADs were applied. Each AD was required to have adequate dosage according to the summary of product characteristics and an adequate duration of at least 4 weeks. Notably, this definition does not weight TRD status for ADs of different classes, augmentation or electroconvulsive therapy, and requires two biological treatments that may be accompanied by psychotherapy.

Symptom severity was measured with MADRS for two-time points of the current major depressive episode (MDE):

1. current MADRS; for the time of study inclusion, when either response was determined or TRD by failing to respond after at least 8 weeks of treatment.
2. retrospective MADRS; for the time of highest symptom load before treatment was initialized for the current MDE.

Treatment response was defined by (1) a current MADRS ≤21 and (2) a decline from retrospective to current MADRS of ≥50%. If response was not achieved after at least two ADs the outcome was labeled as TRD.

The TRD-III sample also comprises patients having undergone a single AD without reaching response, labeled nonresponders. These patients were not considered for this analysis, to avoid confounding from lack of information on response to subsequent treatments, resulting in a sample of 892 individuals (580 female) out of the total 1410 patients included in TRD-III [20].

### Statistical analysis

Stratifying the sample by treatment outcome, generalized linear models (GLM) were computed respectively for age as a numerical and categorical predictor to test for an association with MDE duration (numerical, month), lifetime hospitalization time (numerical, month), lifetime number of MDE (numerical) as well as both retrospective and current MADRS total scores (numerical). Logistic regression was applied to assess the effect of age on treatment outcome (binomial, TRD vs. response). Next to the *p*-values, respective *t*- (linear regression within GLM for age as numerical predictor), *F*- (ANOVA function for GLM for age as categorical predictor), and *z*-statistics (logistic regression for treatment outcome) are reported.

Age was operationalized both as a continuous variable in years and as a categorical variable. Age groups were defined by decades of life-years, resulting in six groups (21–30, 31–40, 41–50, 51‑60, 61‑70, and more than 70). Further, five age groups based on established medical subject headings (MeSH) terms and exploratory clustering results (21–33, 34–48, 49–64, 65‑78, and more than 78 years) were analyzed [21].

Associations with repeated measures (retrospective and current) MADRS items were computed as mixed models provided by the “R” package “nlme” [22]. Thereby, fixed effects included age (as numerical predictor), time point (binomial, retrospective vs. current score) and MADRS item (factor, 10 levels) as well as their three-way interaction. Patient identifier was included as random effect. In case of significant interactions, post hoc models were calculated respectively for time of MADRS assessment or specific items to assess main effects of age.

Finally, items with significant associations within the mixed models were analyzed for differences in item symptom severity operationalized as a binary variable (severe symptoms with score 5–6, vs. low to moderate symptoms with score 0–4). Thereby, logistic regression models with age in years as predictor were built respectively for each MADRS item and time point.

Results were corrected for the number of effects (46; 20 GLM, 12 logistic regression, 14 mixed model) excluding post hoc mixed model tests and exploratory comparison of results for age groups by decade with groups based on MeSH terms. Thus, a corrected *p* threshold of 0.001 was applied. Results not withstanding correction for multiple testing are marked with * throughout the manuscript.

## Results

The mean age in the present TRD-III subsample of 892 patients was 51.19 ± 15.93 years for responders compared to 52.08 ± 13.59 years in TRD. The frequency of female patients was comparable between age groups (58–70.5%, *p* > 0.05). Within patients showing treatment response, no differences in history of MDD regarding age were observed. Within TRD patients, higher age was associated with higher lifetime number of depressive episodes (*p* = 0.002*) and lifetime hospitalization time (*p* < 0.0001). Similar results were found when applying age groups based on MeSH terms. Sample characteristics and comparisons between age groups are further detailed in Table 1, for a comparison with MeSH based age groups please refer to Supplementary Table 1. Prescription rates of antidepressant drug classes are summarized in Supplementary Table 2.

**Table 1.** Sample characteristics stratified by age groups and treatment outcome.

Variable		Age groups						GLM Categorical (Age groups)		GLM Numerical (Life-years)	
		20–30	31–40	41–50	51–60	61–70	> 70				
*n*		71	125	223	238	131	104				
Age		26.3 ± 2.6	35.5 ± 2.9	45.8 ± 2.9	55.4 ± 2.8	64.7 ± 2.9	76.9 ± 5.2	*F*	*p*	*t*	*p*
TRD		33 (46.5%)	75 (60%)	156 (69.7%)	159 (66.8%)	86 (65.7%)	60 (57.7%)	/	0.006* (Chisq)	0.88 (*z*)	n.s.
Sex (female)	TRD	66.7%	68.0%	64.7%	64.8%	64.0%	61.7%	/	n.s.	/	/
	Resp.	68.4%	58.0%	64.2%	65.8%	66.7%	70.5%	/	n.s.	/	/
No. of MDE	TRD	2.6 ± 2.6	3.7 ± 2.3	3.9 ± 2.8	3.8 ± 2.9	4.2 ± 3.1	4.9 ± 3.8	2.5	0.032*	3.14	0.002*
	Resp.	2.2 ± 1.2	3.3 ± 2.9	3.2 ± 2.9	2.8 ± 2.2	3.1 ± 1.9	3.2 ± 2.7	0.84	n.s.	0.82	n.s.
Duration MDE	TRD	8.3 ± 5.1	8.2 ± 5.8	8.6 ± 6.1	9.3 ± 7.0	8.2 ± 7.0	5.9 ± 6.4	2.22	n.s.	−1.37	n.s.
	Resp.	4.6 ± 5.7	5.4 ± 7.1	4.3 ± 5.5	5.9 ± 6.9	5.7 ± 6.4	4.7 ± 5.1	0.52	n.s.	0.43	n.s.
Hosp. time	TRD	2.6 ± 6.1	2.7 ± 7.0	3.9 ± 9.5	5.1 ± 10.3	7.3 ± 11.4	12.4 ± 14.1	8.3	>0.0001	5.82	>0.0001
	Resp.	3.3 ± 4.9	4.7 ± 10.0	5.7 ± 11.	5.05 ± 6.7	3.3 ± 7.0	4.7 ± 9.9	0.63	n.s.	0.06	n.s.
MADRS current	TRD	30.3 ± 6.0	30.6 ± 5.6	30.5 ± 6.0	31.4 ± 6.2	32.4 ± 6.5	32.7 ± 8.3	1.92	n.s.	3.01	0.003
	Resp.	8.6 ± 5.0	8.9 ± 4.8	9.0 ± 5.1	8.2 ± 4.6	8.1 ± 5.3	7.2 ± 4.5	0.92	n.s.	3.00	n.s.
MADRS retrosp.	TRD	34.0 ± 5.5	34.2 ± 5.7	35.0 ± 6.2	36.5 ± 6.6	36.6 ± 7.1	37.8 ± 8.3	3.44	0.005*	4.54	>0.0001
	Resp.	33.3 ± 7.5	31.8 ± 6.9	33 ± 67.3	33.6 ± 8.2	32.3 ± 8.4	31.6 ± 7.3	0.75	n.s.	0.84	n.s.

*Note:* Results of generalized linear models with age as a categorical (*F* and *p*-value) or numerical predictor (*t* and *p*-value) are reported. Results not withstanding correction for multiple testing are marked with *.

Abbreviations: GLM, generalized linear model; MADRS, Montgomery–Åsberg Depression Rating Scale; MDE, major depressive episode; TRD, treatment-resistant depression.

### Treatment resistance and MADRS symptoms

**Figure 1.**
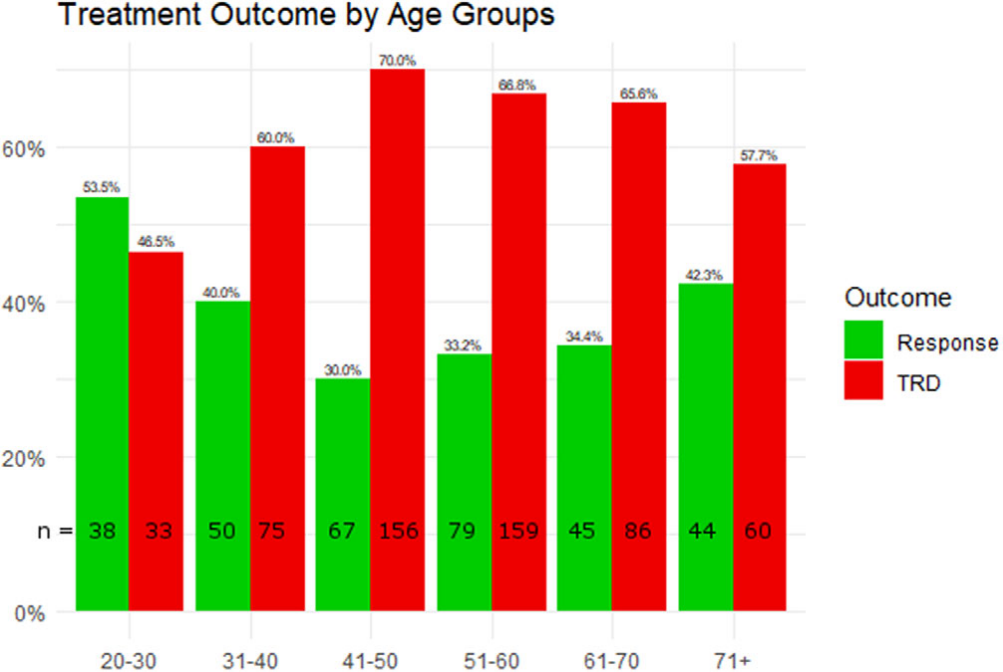
Patients were grouped by age decades (*x*-axis) and treatment outcome (*y*-axis). Percentages of the respective outcomes treatment response and resistant depression (TRD) are plotted, and absolute numbers (*n*) are provided for each subgroup.

Age in years did not predict treatment outcome in the logistic regression model (*p* > 0.05). Despite not withstanding correction for multiple testing, differences in treatment outcome were observed when comparing age groups by either age decades (*p* = 0.007*) or MeSH based categorization (*p* = 0.031*). Youngest patients (21–30 years) showed the lowest rates of TRD (46.48%), followed by patients older than 70 years (57.69%) (Figure 1). Similar patterns of lower rates of resistance in the youngest and oldest age groups (both 52% TRD) compared to patients aged 34–78 years were also observed when applying the MeSH based age categorization.

Total MADRS score was associated with age (*p* < 0.0001) and no interaction with time was observed. Consequently, both the retrospective (*p* < 0.0001) and current (*p* = 0.003*) MADRS scores were associated with age. Assessing MADRS items within the global mixed model, next to main effects of age, item, and time, an interaction effect between age and item was observed in the TRD group (all *p* < 0.0001). No main or interaction effects of age were computed within treatment responders, which were consequently not analyzed further.

Among individual items in TRD, main effects of age were observed for inner tension (*p* = 0.001), reduced appetite (*p* < 0.0001), concentration difficulties (*p* = 0.0002), lassitude (*p* = 0.001), and inability to feel (*p* = 0.03*).

**Figure 2.**
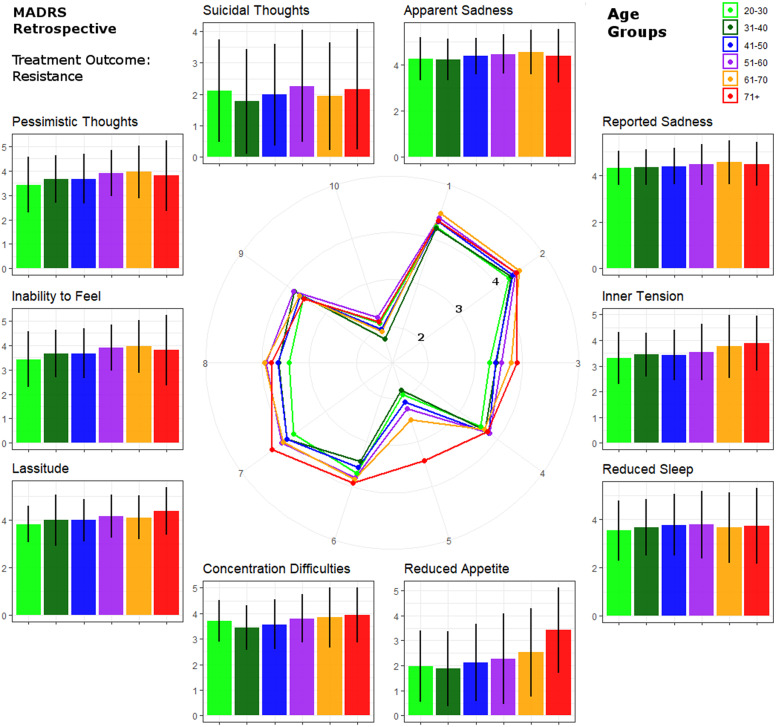
Circular plot of average baseline severity scores of each Montgomery–Åsberg Depression Rating Scale (retrospective MADRS) item within patients with treatment-resistant depression. Scores are provided for age groups ranked by life decades. Mean values and standard errors are provided for each item next to the circular plot for easier interpretation.

Mixed model results are detailed in Table 2. Please also refer to Figure 2 for a graphical representation of retrospective MADRS item scores in TRD. Similar graphics for the current MADRS score in TRD and for responders are presented in the Supplementary Figures.

**Table 2.** Mixed model results.

Model	Effect	DF	*F*	*p*
MADRS total	Age	1/567	17.1	<0.0001
	Time		388.8	<0.0001
	Age × Time		3.5	n.s.
MADRS items	Age	1/567	17.1	<0.0001
	Time	1/10773	510.1	<0.0001
	Item		639.5	<0.0001
	Age × Time		4.6	0.033*
	Age × Item		9.5	<0.0001
	Item × Time		1.3	n.s.
	Age × Time × Item		0.5	n.s.
Post hoc models (stratified by item)				
Inner tension	Age	1/567	10.8	0.001
	Time		124.3	<0.0001
	Age × Time		2.9	n.s.
Reduced appetite	Age	1/567	33.5	<0.0001
	Time		97.4	<0.0001
	Age × Time		4.1	0.045*
Concentration difficulties	Age	1/567	13.9	0.0002
	Time		87.5	<0.0001
	Age × Time		0.33	n.s.
Lassitude	Age	1/567	10.1	0.001
	Time		100.4	<0.0001
	Age × Time		2.6	n.s.
Inability to feel	Age	1/567	4.7	0.03 *
	Time		131.2	<0.0001
	Age × Time		2.3	n.s.

*Note:* Associations not withstanding correction for multiple comparison are marked with *.

Abbreviations: DF, degrees of freedom (numerator/denominator); MADRS, Montgomery–Åsberg Depression Rating Scale.

### Presence of severe symptoms

Following up the mixed model results, severe symptoms indicated by a MADRS item score above 4 were assessed in TRD patients. Associations with age were found for inner tension (*p* = 0.0002 for retrospective MADRS, *p* = 0.001 for current MADRS), reduced appetite (*p* < 0.0001 for retrospective MADRS, *p* = 0.001 for current MADRS), concentration difficulties (*p* < 0.0001 for retrospective MADRS, *p* = 0.0009 for current MADRS) and inability to feel (*p* = 0.0007 for retrospective MADRS, *p* = 0.001 for current MADRS) at both time points while lassitude was linked to age only at retrospective assessment (*p* = 0.0002).

**Figure 3.**
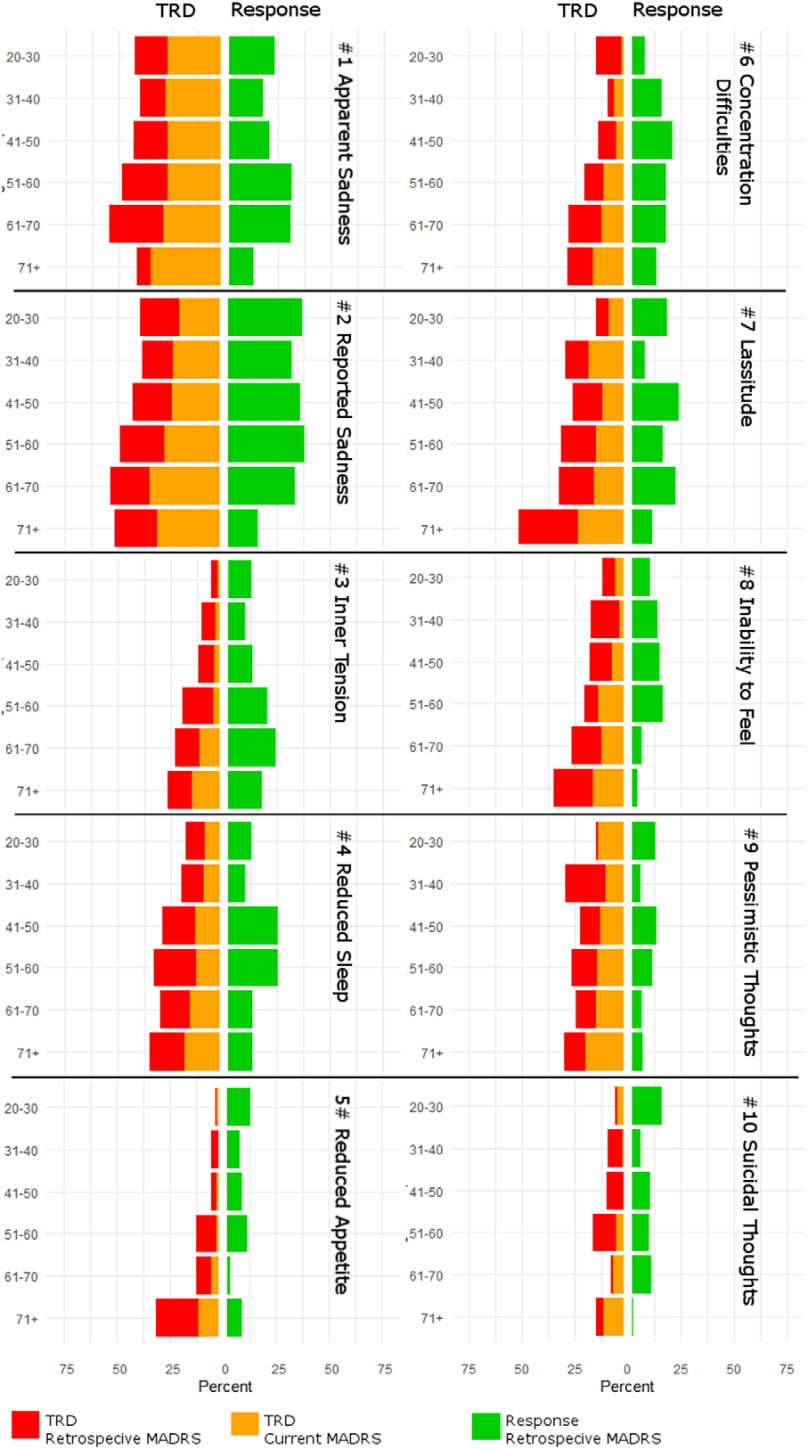
Percentages of patients with severe symptom load, indicated by a Montgomery–Åsberg Depression Rating Scale (MADRS) score of >4 of individual items, within each age group. Patients are stratified by treatment outcome, response, and resistant depression (TRD), and results for baseline and residual symptoms are presented in respective colors.

A graphical depiction of the distributions of severe symptoms among treatment outcome phenotypes and timepoints is provided in Figure 3. Logistic regression results for severe MADRS symptoms are summarized in Supplementary Table 3.

## Discussion

Symptom presentation of TRD was demonstrated to be different between age groups before initiation of treatment, as well as after at least two adequate ADs. While not predicting TRD as treatment outcome phenotype, age is demonstrated to affect symptom severity within TRD.

A role of age in AD treatment outcome had long been suggested, with a focus on late-onset depression and elderly depressed patients. While some clinical studies and a meta-analysis supported efficacy of standard ADs in the elderly [12], another meta-analysis reported limited efficacy of ADs in patients aged over 65 years [23]. Less than half of elderly depressed patients may achieve treatment response and TRD may occur in up to a third of these patients. Consequently, treatment outcome may be substantially worse in the elderly compared to adult patients with MDD, raising concern about the effectiveness of psychopharmacotherapy in these patients [24]. Very high rates of late-life TRD (well beyond 30%) were found in some studies [25], but the criteria used to define TRD greatly differed across studies, and sometimes only required a lack of remission to a single AD trial [26]. In our study, TRD was most common in middle-aged patients, that is, between 40 and 60 years, and TRD frequency did not show a linear increase with age. However, age showed a linear relationship with symptom severity and other unfavorable factors, such as number of MDE in TRD patients. This is in line with the previously drawn observation that older age raises the probability of presenting unfavorable clinical characteristics, associated with severe depression and treatment resistance [27].

Concerning depressive symptom presentation dependent on age, we observed an increase with age in baseline severity as well as residual symptom scores in TRD patients, but not in responders. Earlier studies reported mixed findings regarding the prevalence and severity of depression in old age, but overall they suggested that both decline with age, as subthreshold depressive syndromes become more frequent and confounding factors such as somatic comorbidities more prevalent [6]. While there are hardly reports on the effects of age on symptom severity in TRD specifically [14, 28], our results support increasingly severe symptom load with age in TRD, thereby agreeing with some earlier results on MDD severity in community samples [27].

Specifically, higher rates of reduced sleep, pessimism, psychomotor retardation, and cognitive problems were previously associated with late-life depression [13, 29–31]. In line with these results, in the TRD group age was associated with increased severity of lassitude and concentration difficulties. Lassitude or fatigue was also associated with aging in the general population, implying that depression and particularly TRD may intensify age-related effects [32, 33]. The most robust associations were found for reduced appetite, which has previously been linked to old age depression, specifically in women. On the contrary, increased inner tension with age was reported in men [34], which is in accordance with the concept of male depression [35]. While in this analysis increased scores for both reduced appetite and inner tension were associated with age in TRD, we did not observe sex effects on either of them.

On the other hand, mixed results were reported for core emotional symptoms such as dysphoria and feelings of guilt [29, 30]. Emotional symptoms of depression may be underrepresented in older age groups according to the theorem of “depression without sadness” [36, 37]. Here, no age-related differences were found in TRD regarding sadness as described by MADRS items 1 and 2. However, the inability to feel increased in severity with age in TRD. On a speculative note, persistence of high symptom load (MADRS individual item score > 4) of apparent (83.9%) and reported sadness (61.3%) was most pronounced in the oldest age group in TRD, indicating that these symptoms show resistance to AD treatment specifically in the elderly. This may be indicative of sadness stating an unfavorable prognostic marker in elderly depressed patients.

No significant association with age was found for pessimism and suicidality. Higher risk for suicide and fatal outcomes in elderly depressed patients has been reported [6], while rates of suicidal ideation were not generally elevated in older age groups [38]. Contrasting these findings, higher symptoms of suicidality in younger compared to older MDD patients have been shown in a nationwide cohort study in Korea [39]. In synopsis, our findings do not suggest age as a predictor for suicidality or pessimism.

However, the context of the TRD-III data set must be considered when interpreting these results. We present results from an observational, cross-sectional, naturalistic study that cannot be directly compared to controlled clinical trials. Randomized controlled trials (RCT) are the gold-standard of scientific hypothesis testing and allow for much more stringent comparisons. On the other hand, controlled trials on a severe disorder such as TRD that requires urgent treatment are difficult to realize and recent population-based data suggest that most psychiatric patients would not meet typical eligibility criteria and thus may never be represented by current gold-standard studies [40]. Open naturalistic studies with a defined treatment protocol, such as STAR*D [41] or a German study specifically addressing treatment response to escitalopram in elderly [42], have the advantage of representativeness but offer a straightforward evaluation of treatment outcome compared to the GSRD studies that comprise patients with a plethora of past and active treatments. While the real-world clinical representativeness is also a strength of our study, the broad spectrum of different treatment protocols based on clinical judgment hindered assessment of effects of specific AD agents. The observed prescription patterns followed established recommendations for TRD in the elderly as mirtazapine treatment as well as augmentation with atypical antipsychotics were most frequently prescribed in patients above 60 years of age while tricyclic antidepressants (TCA) were least common in this age group (3.85%) [13, 15, 43]. In summary, caveats such as clinical heterogeneity and self-selection bias must be considered for open naturalistic studies.

Further, similarly to personality disorders, severe neurological disorders primary to MDD were considered as exclusion criteria. This protocol may have affected age groups differently as neurodegenerative disorders become more frequent with age, resulting in sampling among healthier cohorts of elderly patients compared to the general elderly population. On the other hand, mild cognitive impairment was not assessed clinically and may interfere with depressive symptoms observed in elderly patients.

However, an important limitation of this analysis stems from retrospective assessment of baseline MADRS scores due to the cross-sectional nature of the study. Consequently, assessment of baseline symptom severity was dependent on patients´ recollection and medical documentation. There is evidence that short-term retrospective symptom assessment may reflect the actual symptom load in depression [44]. Recall of core depressive symptoms showed high accuracies around 90% for depressive episodes up to 2 years past. However, the study by Dunlop et al. only investigated recall of symptom types but not intensities [44]. Considering the known association between depression and functional memory alterations [45], overestimation of past symptoms by TRD patients compared to treatment responders may bring bias to our results. However, in our study, comparable item scores for retrospective MADRS between treatment responders and resistant patients (*p* > 0.05) were observed which can be indicative for a correct recollection of symptomatology. While it does not seem that TRD patients overestimate their baseline symptoms compared to treatment responders, we cannot rule out that the retrospective symptom assessment is biased by poor or distorted recollection.

Finally, this analysis ranks among a broad range of investigations within the framework of the GSRD and the TRD data sets [10]. Given that this is the only study specifically analyzing the effects of age on depressive symptoms and disease characteristics, we did not account for previous statistical tests on other research questions assessed earlier by the GSRD in the same data set. While this approach follows common scientific practice [46], we cannot rule out false-positive findings despite applying a *p* threshold of 0.001 corrected for the tests performed in this analysis.

In summary, the TRD-III sample is suitable to investigate effects of age in a continuum of severe, long-standing MDD rather than address specific phenotypes suggested by earlier work, such as late life, late onset, vascular or executive-dysfunction depression [47]. Only 11.7% of the sample was older than 70 years, which contributed to the decision to analyze effects of age throughout the whole range of life rather than targeting depression in the elderly. Regarding late-onset depression, 25.9% of patients aged 60–70 and 49.5% of patients beyond 70 years of age reported no episodes before the age of 60. While late-onset depression showed favorable response rates of 41.6% compared to 35.6% of earlier onset patients, the gap almost closed when considering only patients with recurrent depression (36.1 vs. 34.4%). In terms of severity and symptom presentation, early and late-onset depression were previously shown to be similar when the number of episodes was comparable, despite variations of other characteristics such as personality and family history [48, 49]. Along these lines, in a longitudinal observation study in elderly MDD patients, being depressed 2 years after baseline was associated with earlier onset and chronicity of illness [50]. Recurrent depression and the number of previous episodes may therefore be more relevant for the risk of TRD than early compared to late-onset depression [17, 18, 51].

In synopsis, the general symptom load as well as specific symptoms such as appetite loss, inner tension, inability to feel, and concentration difficulties were shown to increase with age. While we did not find elevated rates of TRD in elderly depressed patients, other studies with more stringent treatment protocols point toward lower efficacy of AD treatment across age groups. Higher rates of treatment nonresponse were observed in patients beyond 75 years of age in a large German study on escitalopram [42]. Taking together these results, TRD may be more common but also more concerning in elderly patients with MDD. The increase of symptom severity with age demonstrated in the group with TRD, both before initiation of treatment as well as after adequate treatment, is further emphasized by world populations ever growing older. Potentially, more recently established agents such as ketamine will further remedy symptoms specifically associated with TRD in old age such as cognitive problems, however, preliminary results did not fully support this claim [52]. Thus, our data underline the need for precision medicine and tailored treatment algorithms that take in account age groups as well as relevant clinical variables associated therewith, such as the number of episodes, chronicity, and symptom severity.

## Data Availability

Data are available from the corresponding author upon reasonable request.

## Supplementary material

For supplementary material accompanying this paper visit http://doi.org/10.1192/j.eurpsy.2023.17. click here to view supplementary material
