# Supplementary material for: Age as a moderating factor of treatment resistance in depression
Source: Eur Psychiatry. 2023 Apr 20;66(1):e35. doi: 10.1192/j.eurpsy.2023.17 (PMC10228354; doi:10.1192/j.eurpsy.2023.17)
Supplement: Supplementary file 1 [file S0924933823000172sup001.docx]

| **Variable** | | **Age Groups** | | | | | **GLM**  **Categorical**  **(Age Groups)** | |
| --- | --- | --- | --- | --- | --- | --- | --- | --- |
|  |  | **20-33** | **34-48** | **49-64** | **65-78** | **> 78** |  |  |
| n | | 105 | 263 | 359 | 136 | 29 |  |  |
| Age | | 28.1 ± 3.4 | 41.9 ± 4.2 | 55.9 ± 4.3 | 71.2 ± 3.9 | 83.7 ± 4.2 | **F** | **p** |
| Outcome (TRD) | | 52.4% | 66.2% | 67.1% | 61.8% | 51.7% | / | 0.038*  (Chisq) |
| Sex (female) | TRD | 67.3 | 66.1 | 65.6 | 60.7 | 53.3 | / | n.s. |
|  | Resp. | 68.0 | 58.4 | 67.8 | 69.2 | 64.3 | / | n.s. |
| Nr. of  MDE | TRD | 2.7 ± 2.1 | 3.9 ± 2.8 | 3.8 ± 2.8 | 4.7 ± 3.6 | 4.9 ± 4.1 | 3.6 | 0.006* |
|  | Resp. | 2.1 ± 1.1 | 3.4 ± 3.1 | 3.0 ± 2.4 | 3.1 ± 2.2 | 2.7 ± 2.8 | 1.9 | n.s. |
| Duration  MDE | TRD | 8.6 ± 5.3 | 8.1 ± 5.7 | 9.1 ± 6.9 | 6.7 ± 6.6 | 7.3 ± 8.2 | 2.16 | n.s. |
|  | Resp. | 5.1 ± 6.3 | 4.7 ± 6.3 | 5.3 ± 6.4 | 5.4 ± 5.9 | 4.3 ± 4.1 | 0.15 | n.s. |
| Hosp.  Time | TRD | 1.8 ± 4.9 | 3.6 ± 9.2 | 5.4 ± 10.4 | 10.6 ± 13.9 | 9.8 ± 9.2 | 8.81 | > 0.0001 |
|  | Resp. | 3.6 ± 6.7 | 5.2 ± 10.2 | 5.0 ± 7.9 | 4.6 ± 9.8 | 1.9 ± 2.7 | 0.67 | n.s. |
| MADRS  Current | TRD | 29.9 ± 5.5 | 30.4 ± 5.9 | 31.6 ± 6.3 | 31.9 ± 6.7 | 35.2 ± 10.1 | 3.25 | 0.012* |
|  | Resp. | 8.5 ± 4.9 | 9.1 ± 5.0 | 8.2 ± 4.7 | 7.2 ± 4.9 | 8.6 ± 4.5 | 1.26 | n.s. |
| MADRS  Retrosp. | TRD | 34.0 ± 5.5 | 34.2 ± 6.0 | 36.9 ± 6.6 | 36.3 ± 7.5 | 40.1 ± 9.2 | 6.85 | > 0.0001 |
|  | Resp. | 33.1 ± 7.5 | 32.6 ± 7.0 | 33.8 ± 7.9 | 31.5 ± 7.7 | 30.3 ± 9.0 | 1.35 | n.s. |

**Supplementary Table 1.** Sample characteristics stratified by age groups (MeSH terms) and treatment outcome. Results of GLMs with age as a categorical (F & p value) or numerical predictor (t & p value) are reported. Abbreviations: MADRS = Montgomery Åsberg Depression Rating Scale; MDE = major depressive episode; GLM = generalized linear model

| **Variable** | **Age Groups** | | | | | | |
| --- | --- | --- | --- | --- | --- | --- | --- |
|  | **20-30** | **31-40** | **41-50** | **51-60** | **61-70** | **> 70** | **Total** |
| n | 71 | 125 | 223 | 238 | 131 | 104 | 892 |
| Sex (female) | 48 (67.6%) | 80 (64%) | 144 (67.6%) | 155 (64.6%) | 85 (64.9%) | 68 (65.4%) | 580 (65%) |
| **Antidepressants** | | | | | | | |
| TCA | 3 (4.2%) | 11 (8.8%) | 17 (7.6%) | 25 (10.5%) | 13 (9.9%) | 4 (3.9%) | 73 (8.2%) |
| SSRI | 41 (57.8%) | 71 (56.8%) | 117 (52.5%) | 125 (52.5%) | 76 (58%) | 64 (61.5%) | 494 (55.4%) |
| SNRI / NDRI | 15 (21.1%) | 38 (30.4%) | 70 (31.4%) | 80 (33.6%) | 45 (34.4%) | 30 (28.9%) | 278 (31.2%) |
| Mirtazapine | 12 (16.9%) | 8 (6.4%) | 37 (16.6%) | 51 (21.4%) | 27 (20.6%) | 40 (38.5%) | 175 (19.6%) |
| Typical AP | **/** | 3 (2.4%) | 9 (4.04%) | 11 (4.6%) | 6 (4.6%) | 10 (9.6%) | 39 (4.4%) |
| Atypical AP | 13 (18.3%) | 31 (24.8%) | 60 (26.9%) | 74 (31.1%) | 50 (38.2%) | 37 (35.6%) | 265 (29.7%) |
| Lithium | 4 (5.6%) | 4 (3.2%) | 7 (3.1%) | 15 (6.3%) | 5 (3.8%) | 7 (6.7%) | 42 (4.7%) |

**Supplementary Table 2.** Sample characteristics with means (standard deviations) for numerical variables and counts (percentages) for factors. Relevant clinical and sociodemographic variables are presented ordered by age groups.

Abbreviations: AP = antipsychotic, n = number, NDRI = noradrenaline-dopamine reuptake inhibitors, SNRI = serotonin-norepinephrinereuptake inhibitors, SSRI = selective serotonin reuptake inhibitors, TRD = treatment resistant depression

| **Model** | **MADRS** | **z** | **p** |
| --- | --- | --- | --- |
| Inner Tension | Retrospective | 3.7 | 0.0002 |
|  | Current | 3.2 | 0.0014 |
| Reduced Appetite | Retrospective | 5.6 | < 0.0001 |
|  | Current | 3.2 | 0.0012 |
| Concentration Difficulties | Retrospective | 3.9 | < 0.0001 |
|  | Current | 3.3 | 0.0009 |
| Lassitude | Retrospective | 3.7 | 0.0002 |
|  | Current | 1.4 | n.s. |
| Inability to Feel | Retrospective | 3.4 | 0.0007 |
|  | Current | 3.2 | n.s. |

**Supplementary Table 3.** Logistic regression results for age as a predictor of dichotomized MADRS items (score > 4 indicating severe symptom load). Abbreviations: MADRS = Montgomery Åsberg Depression Rating Scale


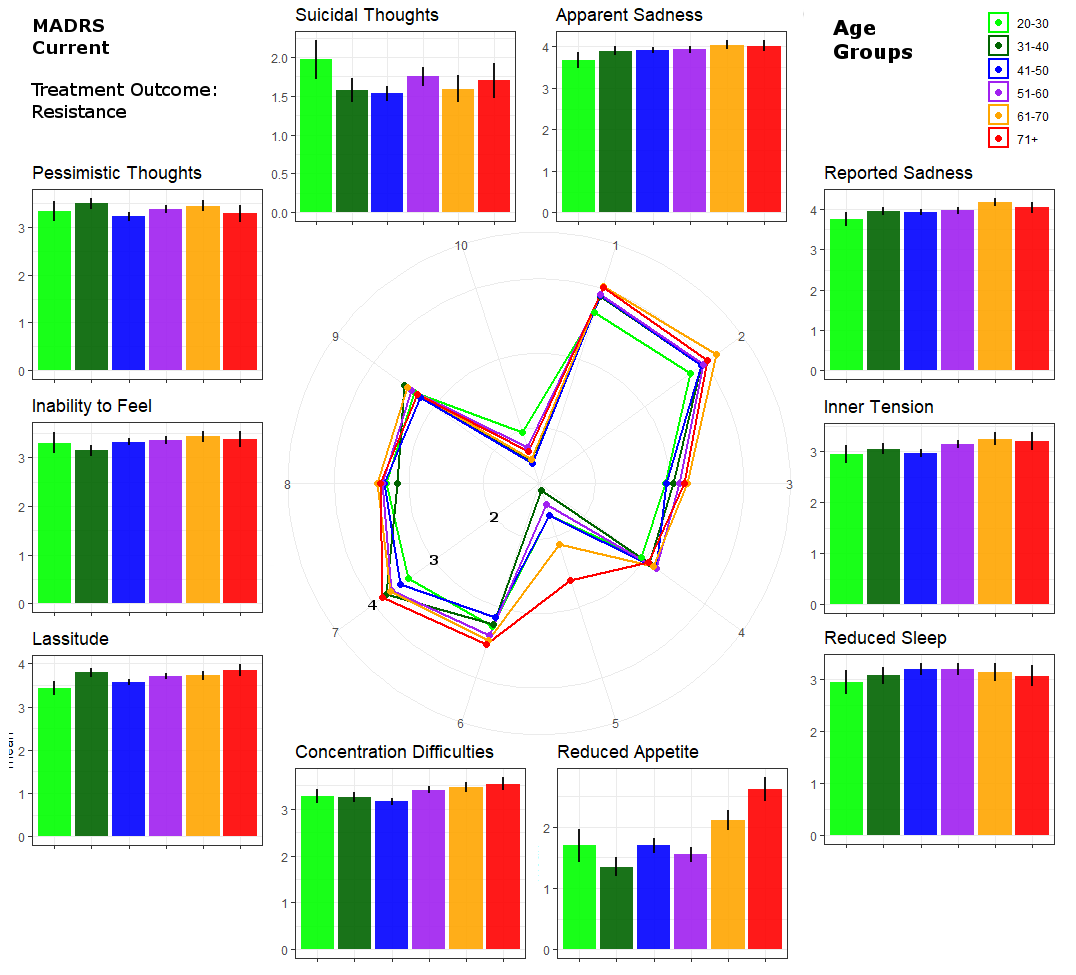


**Supplementary Figure 1.** Circular plot of average residual severity scores of each Montgomery-Åsberg depression rating scale (MADRS) item within patients showing treatment resistance. Scores are provided for age groups ranked by life decades. Mean values and standard errors are provided for each item next to the circular plot for easier interpretation.

**
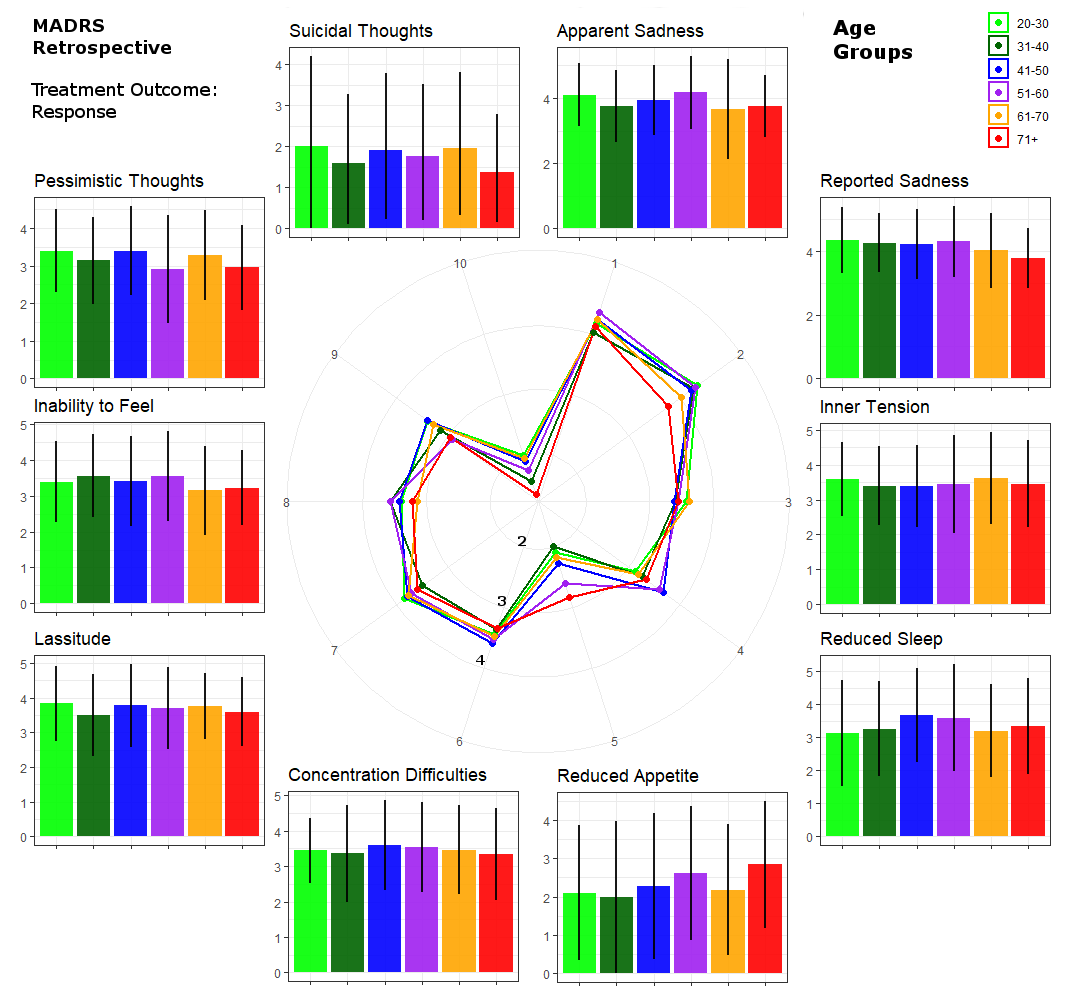
**

**Supplementary Figure 2.** Circular plot of average baseline severity scores of each Montgomery-Åsberg depression rating scale (MADRS) item within patients showing treatment response. Scores are provided for age groups ranked by life decades. Mean values and standard errors are provided for each item next to the circular plot for easier interpretation.

**
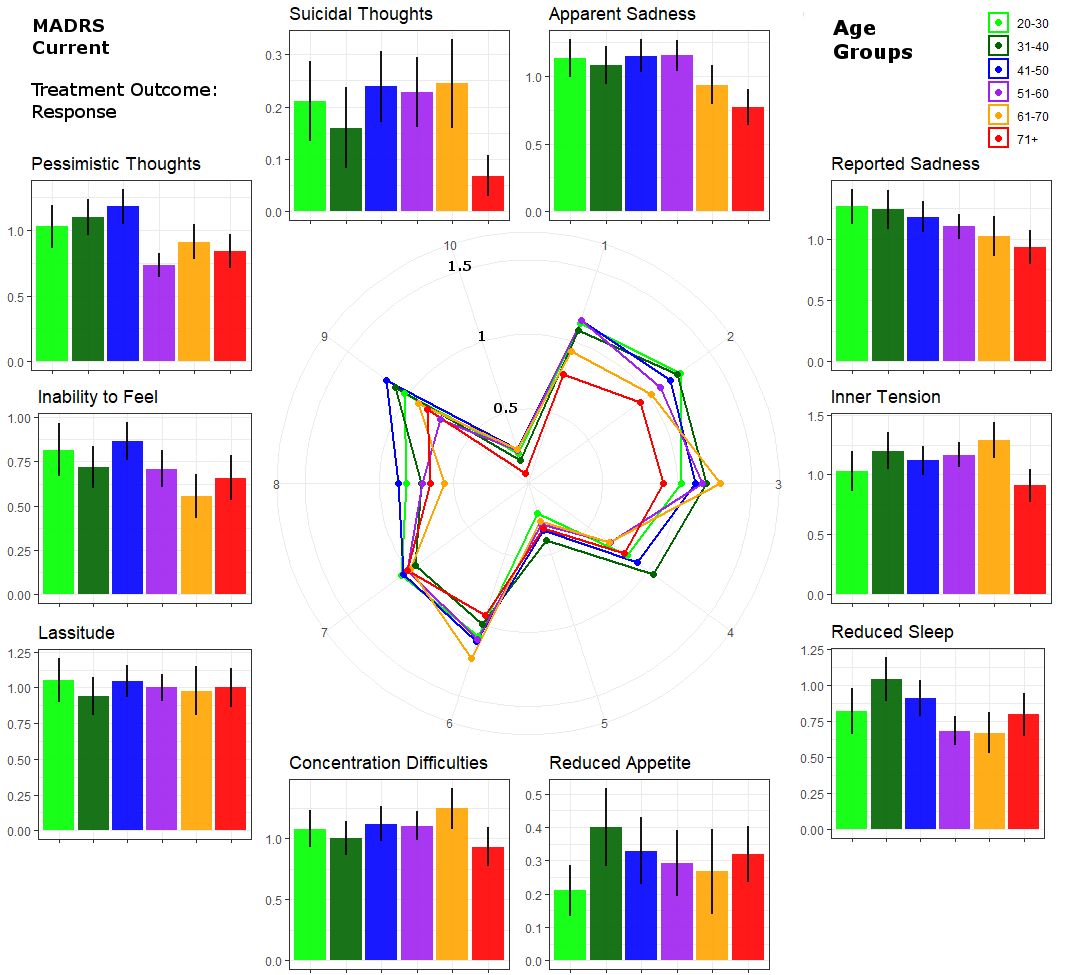
**

**Supplementary Figure 3.** Circular plot of average residual severity scores of each Montgomery-Åsberg depression rating scale (MADRS) item within patients showing treatment response. Scores are provided for age groups ranked by life decades. Mean values and standard errors are provided for each item next to the circular plot for easier interpretation.
